# Regulation of Tau Alternative Splicing: A Novel Role for the Ribonucleoprotein RBM20

**DOI:** 10.3390/ijms27094001

**Published:** 2026-04-29

**Authors:** Andrea Corsi, Angela Valentino, Maria Giusy Bruno, Giacomo Menichetti, Francesca Belpinati, Marta P. Pereira, Maria Teresa Valenti, Alessandra Ruggiero, Elisabetta Trabetti, Cristina Bombieri, Maria Grazia Romanelli

**Affiliations:** 1 Department of Neuroscience, Biomedicine and Movement Sciences, University of Verona, 37134 Verona, Italy; andrea.corsi@univr.it (A.C.); angela.valentino@univr.it (A.V.); mariagiusy.bruno@univr.it (M.G.B.); giacomo.menichetti@univr.it (G.M.); francesca.belpinati@univr.it (F.B.); mariateresa.valenti@univr.it (M.T.V.); alessandra.ruggiero@univr.it (A.R.); elisabetta.trabetti@univr.it (E.T.); 2 Departamento de Biología Molecular, Instituto Universitario de Biología Molecular (IUBM) and Centro de Biología Molecular Severo Ochoa (CBMSO), Universidad Autónoma de Madrid, 28049 Madrid, Spain; m.pereira@uam.es

**Keywords:** MAPT, Tau, PTBP1, RBM20, alternative splicing

## Abstract

Tau is a protein associated with microtubules principally expressed in neuronal cells, where it plays a fundamental role in cytoskeleton stabilization and axonal transport. Several diseases collectively named tauopathies, such as Alzheimer’s disease, have been associated with an imbalance in the expression of alternative spliced Tau transcripts and the accumulation of hyperphosphorylated Tau, causing dysfunction and death of neuronal cells. Therefore, understanding the Tau exon splicing mechanisms may contribute to elucidating molecular factors that could underlie the development of neurodegenerative disorders. The aim of this study was to define the role of selected splicing factors in regulating Tau exon expression in cell lines and neuronal organoids. We demonstrated the role of the RNA-binding motif protein 20 (RBM20) splicing factor in regulating Tau exon 6 and exon 10, applying RNA-binding assay and qPCR analyses. Furthermore, we demonstrated that Tau expression was regulated during cerebral organoid differentiation, recapitulating in vivo Tau expression. These results suggest the feasibility of using brain organoid technology to study Tau alternative splicing during neural development, confirming that 3D cellular models could be used to study and characterize pathological processes taking place in Tau-related pathologies.

## 1. Introduction

Tau is a microtubule-binding protein encoded by the Microtubule Associated Protein Tau (*MAPT*) gene. It is highly expressed in the central nervous system (CNS) and peripheral nervous system (PNS), where it participates in the assembly and stability of microtubules and supports axonal transport in neurons [1]. Tau dysfunction has been closely linked to neurodegenerative conditions characterized by abnormal Tau hyperphosphorylation and aggregation, resulting in intracellular inclusions and progressive neuronal loss [2].

The *MAPT* gene is located on chromosome 17, and it is organized into 16 exons that are extensively regulated by alternative splicing [3]. Exons 2, 3, 4a, 6, 8, and 10 are subject to alternative splicing events resulting in the production of more than a dozen different Tau protein isoforms, potentially bearing different functions [4].

Tau protein structure can be subdivided into three major domains: N-terminal projection region (NTR), proline-rich motif (PRR), and C-terminal microtubule-binding domain (MTBD). The NTR spans from the beginning of the Tau sequence to the first amino acids of exon 7, and the PRR spans from exon 7 to exon 9, while MTBD is codified from the last part of exon 9 to exon 12. In particular, the C-terminal region is characterized by highly conserved repeated fragments known as R1, R2, R3, and R4. These repetitive peptide sequences, through the interaction with tubulin, promote the stability and the assembly of microtubules and regulate axonal transport [4,5].

The six different Tau protein isoforms can be classified based on alternative splicing of exons 2 and 3, which generates 0N, 1N, or 2N isoforms, and on the number of repeats in the MTBD: retention or skipping of exon 10 produces 4R-Tau or 3R-Tau, respectively [5]. The imbalance of Tau 3R and 4R exon 10 isoforms has been linked to Alzheimer’s Disease (AD), Parkinson’s Disease (PD), and Frontotemporal dementia and Parkinsonism linked to chromosome 17 (FTDP-17) [6]. In these disorders, Tau is abnormally phosphorylated and misfolded, resulting in the accumulation of cytoplasmic fibrillary tangles, which lead to neuronal death [6,7]. Collectively, neurodegenerative disorders, sharing the pathological hallmark of Tau misfolding, aggregation, and accumulation in the brain, are referred to as tauopathies [8]. Furthermore, exon 6-containing Tau isoforms have been demonstrated to block Tau polymerization [9] and promote neural differentiation in vitro [10].

As the disruption of the balance between different Tau isoforms appears to be a common pathological hallmark of tauopathies [11], understanding the molecular mechanisms underlying Tau alternative splicing regulation is pivotal to designing novel therapeutic approaches to normalize Tau expression in neurodegenerative diseases. In this study, we focused on the role of selected RNA-binding proteins (RBPs) in regulating Tau isoform expression. RBPs act as *trans*-acting factors, able to recognize RNA *cis*-acting sequences, influencing the splicing process [12,13]. We chose to analyze the role of polypyrimidine-binding tract protein 1 (PTBP1) and RBM20 splicing factors in regulating Tau exon 6 and 10 based on the presence of their putative consensus binding sequences in the exon flanking regions.

PTBP1 is an abundant and widely expressed RBP, whose main function is the regulation of alternative splicing by promoting exon exclusion [14,15]. While PTBP1 is ubiquitously expressed, the level of its expression is modulated in a tissue-specific manner, as described during cardiomyocyte differentiation [16], cerebral cortex development [17], and B-cell selection in the germinal center [18]. PTBP1 participates in the maintenance of stemness in several cell types and is required during embryonic development [19]. Furthermore, PTBP1 is of particular importance in neural development, as it participates in axon formation, synapse formation, and neuronal apoptosis [20]. Indeed, PTBP1 knockdown induces the formation of new functional neurons in both human brain organoids and in vivo mouse brains [21].

RBM20 is a tissue-specific RBP, mainly expressed in cardiac skeletal muscle [14,22], that regulates alternative splicing of several proteins involved in pathways fundamental to heart function, including sarcomere assembly, ion transport, and diastolic function [23,24,25]. PTBP1 and RBM20 are both involved in heart development and pathology, participating in alternative splicing of genes fundamental for heart function, such as Calcium Voltage-Gated Channel Subunit α1C (CACNA1C) [26], Formin homology 2 domain containing 3 (FHOD3) [27], and Titin (TTN) [23,26,28]. Recently, it has been demonstrated that RBM20 plays a role in neuronal mRNA splicing regulation, suggesting a new tissue-specific role for an RBP historically associated with cardiac tissue [29]. In the present study, applying minigenes, brain organoids, and neuronal cell line analyses, we collected evidence of a novel role of RBM20 in Tau splicing regulation.

## 2. Results

### 2.1. Tau Exons 6 and 10 Are Cell Type Differentially Expressed

**Figure 1:**
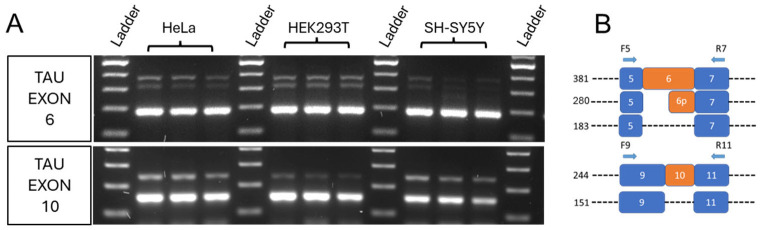
Analysis of Tau exons 6 and 10 alternative splicing in human HeLa, HEK293T, and SH-SY5Y cell lines. (**A**) Electrophoretic run of RT-PCR reaction showing Tau exon 6 and 10 isoforms produced by alternative splicing. (**B**) Scheme depicting the primers and strategy used for PCR amplification. F5 = Forward primer annealing on Tau exon 5; R7 = Reverse primer annealing on Tau exon 7; F9 = Forward primer annealing on Tau exon 9; R11 = Reverse primer annealing on Tau exon 11.

The six major Tau isoforms are referred to as 0N, 1N, or 2N based on the alternative splicing of the N-terminal exons, while alternative splicing of the C-terminal region gives rise to 3R or 4R Tau isoforms. Exon 6 regulation is expected to generate multiple transcripts depending on its inclusion or exclusion (called respectively 6+ and 6−), and the use of internal splice sites can also produce additional isoforms, such as 6p and 6d variants that encode truncated Tau proteins lacking the MTBD, whereas exon 10 regulation generates 3R-Tau or 4R-Tau [1]. First, we compared exon 6 and exon 10 expression in three different cell lines: HeLa, HEK293T, and SH-SY5Y. Three exon 6 isoforms, corresponding to 6+, 6p, and 6− were observed in all three cell lines, as well as the two expected exon 10 isoforms corresponding to Tau 4R and Tau 3R (Figure 1A). Primers were designed to anneal the flanking constitutive exons (5, 7, 9, and 11), which are invariably included in the mature transcript (Figure 1B). The results showed that isoforms excluding exons 6 and 10 represent the predominant variants in all three cell lines. These preliminary results confirmed that the splicing factors required for Tau alternative splicing regulation are components of the molecular splicing machinery in all three cell lines and allowed us to utilize these cell line models for further analyses.

### 2.2. FIMO Bioinformatic Analysis Identifies Putative RBP Binding Sites in Genomic Regions Surrounding Tau Exons 6 and 10

RBM20 has been shown to function in a tissue-specific manner, often competing with PTBP1 in regulating exon splicing [25,26]. Based on this evidence, we hypothesized that RBM20 can contribute to the regulation of *MAPT* exons. We used the FIMO web tool to scan *MAPT* genomic regions encompassing exons 6 and 10 for putative PTBP1 and RBM20 binding sites (motif selection criteria are described in the Materials and Methods Section 4.1) to map regulatory regions to be cloned into a minigene tool useful for testing Tau exon-skipping regulation.

**Figure 2:**
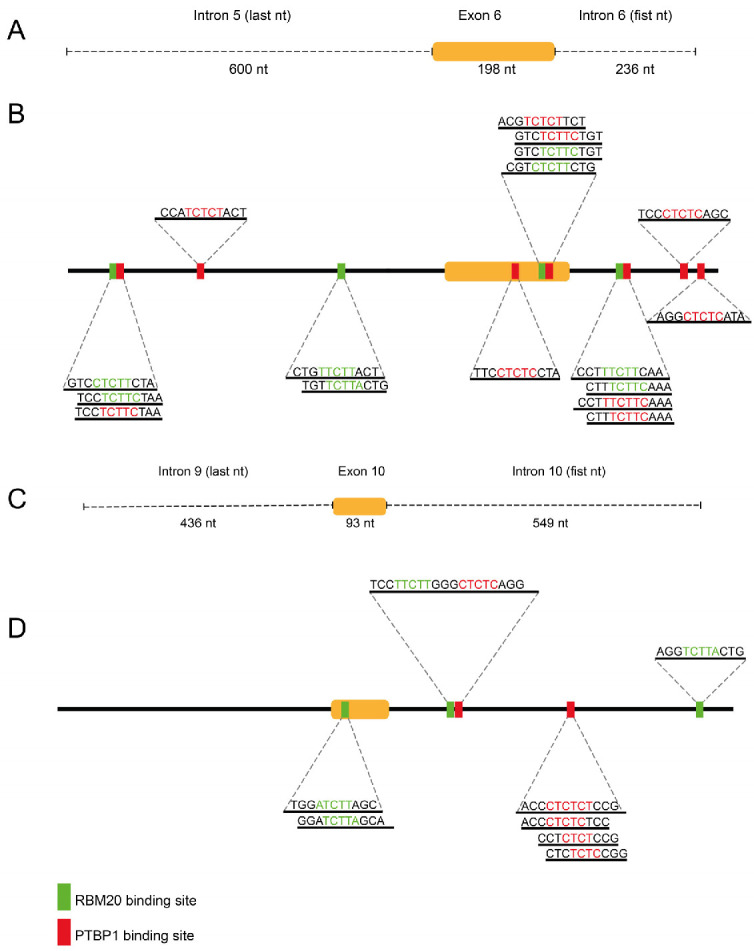
Putative PTBP1 and RBM20 binding sites in Tau exon 6 and 10 regions revealed via bioinformatic analysis. Schematic representation of the *MAPT* genomic region surrounding exon 6 (**A**) and exon 10 (**C**) scanned using the FIMO web tool. Detailed representation of the putative binding sites within exon 6 (**B**), exon 10 (**D**), and their flanking introns, identified via FIMO scanning. Exons 6 and 10 are indicated as orange boxes. Binding sites for RBM20 are represented in green, while binding sites for PTBP1 are represented in red.

A 1035 nt sequence comprising the last 601 nt of intron 5, exon 6, and the first 236 nt of intron 6 was analyzed with FIMO (Figure 2A). Using a *p*-value threshold of 0.001, eight putative RBM20 binding sites and nine putative PTBP1 binding sites were identified (Figure 2B).

For *MAPT* exon 10, a 1078 nt region including the last 436 nt of intron 9, exon 10, and the first 549 nt of intron 10 was scanned (Figure 2C). Four putative RBM20 binding sites and five putative PTBP1 binding sites were detected in the selected sequence (Figure 2D).

The genomic regions encompassing exons 6 and 10 were cloned into the RHCglo vector [30], a well-established system for minigene splicing assays. The resulting plasmids were designated pRHCglo-MAPT-E6 (minigene T6; Figure S1) and pRHCglo-MAPT-E10 (minigene T10; Figure S2).

### 2.3. PTBP1 and RBM20 Overexpression Induces Tau Exons 6 and 10 Exclusion

**Figure 3:**
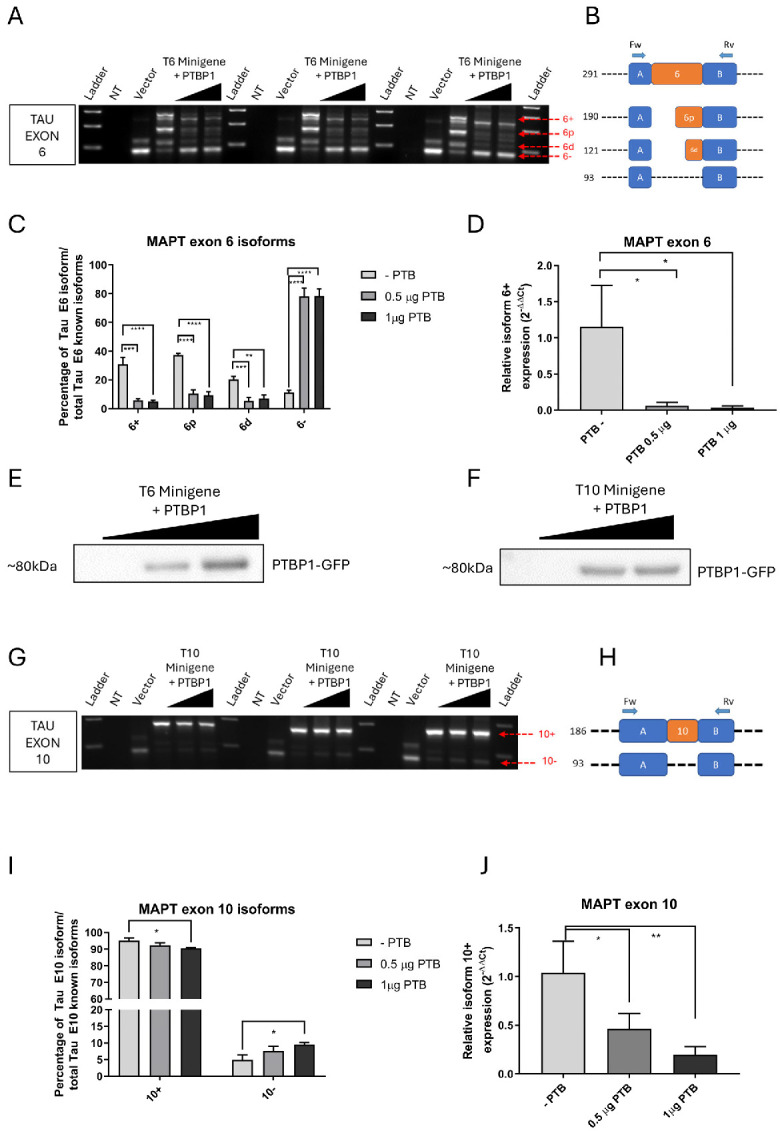
Analysis of Tau exon 6 and 10 regulation by minigene constructs under PTBP1 overexpression conditions. (**A**,**G**) Electrophoretic gel analysis of RT-PCR products showing alternative splicing of Tau exon 6 and exon 10, respectively. (**B**,**H**) Scheme depicting the four exon 6 isoforms, the two exon 10 isoforms, and their relative length in bp, respectively. (**C**,**I**) Densitometric analysis of the RT-PCR products of exon 6 and exon 10 isoforms, after gel electrophoresis. (**D**,**J**) qPCR analysis showing the reduction in Tau exon 6+ and exon 10 isoforms, respectively. (**E**,**F**) WB image showing an increase in PTBP1-GFP production after transfection with the PTBP1-GFP expression vector. <.NT = non-transfected cells; Vector = RHC-glo transfected cells; * = *p*-value ≤ 0.05; ** = *p*-value ≤ 0.01; *** = *p*-value ≤ 0.001; **** = *p*-value ≤ 0.0001.

To highlight the involvement of PTBP1 and RBM20 in the expression of Tau exons 6 and 10, we analyzed the splicing patterns induced by overexpression of these RBPs in the presence of T6 and T10 minigene constructs transfected into HEK293T cells. As revealed by electrophoretic separation, the T6 construct generated four splicing isoforms: a fully included isoform (6+, 291 bp), two partially included isoforms arising from alternative 3′ splice sites (6p, 190 bp; 6d, 121 bp), and a fully excluded isoform (6−, 93 bp) (Figure 3A,B). Densitometric quantification demonstrated a statistically significant reduction in the 6+, 6p, and 6d isoforms upon PTBP1 overexpression compared with the control. Conversely, the 6− isoform was significantly increased (Figure 3C). A qPCR assay validated these RT-PCR findings, confirming a statistically significant reduction in the exon 6+ isoform in the PTBP1-overexpressed samples (Figure 3D).

The minigene-splicing assay was also performed using minigene T10. Two isoforms were detected: an exon-included isoform (10+, 186 bp) and an exon-skipped isoform (10−, 93 bp) (Figure 3G,H). Results from densitometric analysis revealed a significant reduction in the 10+ isoform and a corresponding increase in the 10− isoform at the highest level of PTBP1 overexpressing vector transfection, relative to control (Figure 3I). Both the RT-PCR and qPCR assays showed a significant reduction in the exon 10+ isoform upon PTBP1 overexpression (Figure 3J). Western blot analysis validated PTBP1 overexpression (Figure 3E,F). Overall, these results suggest that increased amounts of PTBP1 can affect Tau exon 6 and 10 exclusion by recognizing *cis*-acting elements within the flanking intron regions.

**Figure 4:**
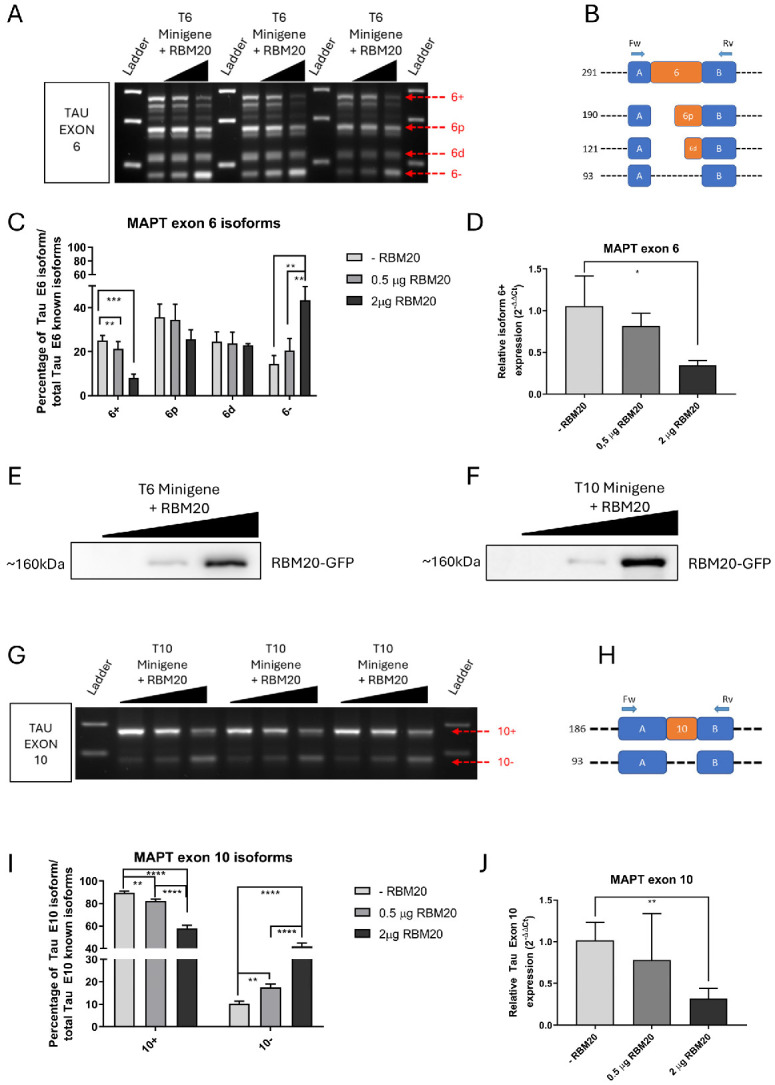
Evaluation of Tau exon 6 and 10 regulation by the use of minigene constructs in the presence of RBM20 overexpression. (**A**,**G**) Electrophoretic gel analysis of RT-PCR products showing alternative splicing of Tau exon 6 and exon 10, respectively. (**B**,**H**) Scheme depicting the four exon 6 isoforms, the two exon 10 isoforms, and their relative length in bp, respectively. (**C**,**I**) Densitometric analysis of RT-PCR products of exon 6 and exon 10 isoforms after gel electrophoresis. (**D**,**J**). qPCR analysis showing the reduction in Tau exon 6+ and exon 10 isoforms, respectively. (**E**,**F**) WB image showing an increase in RBM20-GFP production after transfection with the RBM20-GFP expression. * = *p*-value ≤ 0.05; ** = *p*-value ≤ 0.01; *** = *p*-value ≤ 0.001; **** = *p*-value ≤ 0.0001.

Similar analyses were performed in the presence of overexpression of the RBM20 protein. Electrophoretic analysis revealed that RBM20 overexpression strongly reduced exon 6 inclusion (Figure 4A,B). Densitometric quantification confirmed a statistically significant decrease in the 6+ isoform upon RBM20 overexpression relative to control (Figure 4C), which was validated by qPCR (Figure 4D). Western blot analysis confirmed RBM20 overexpression (Figure 4E). In the presence of T10 minigene, RBM20 overexpression induced a reduction in exon 10 inclusion (Figure 4G–I), confirmed by qPCR (Figure 4J). These findings suggest that RBM20 can modulate Tau exon 6 and 10 splicing by promoting exon exclusion through *cis*-regulatory sequences located within the flanking intronic regions.

### 2.4. PTBP1 and RBM20 Bind Tau mRNA

**Figure 5:**
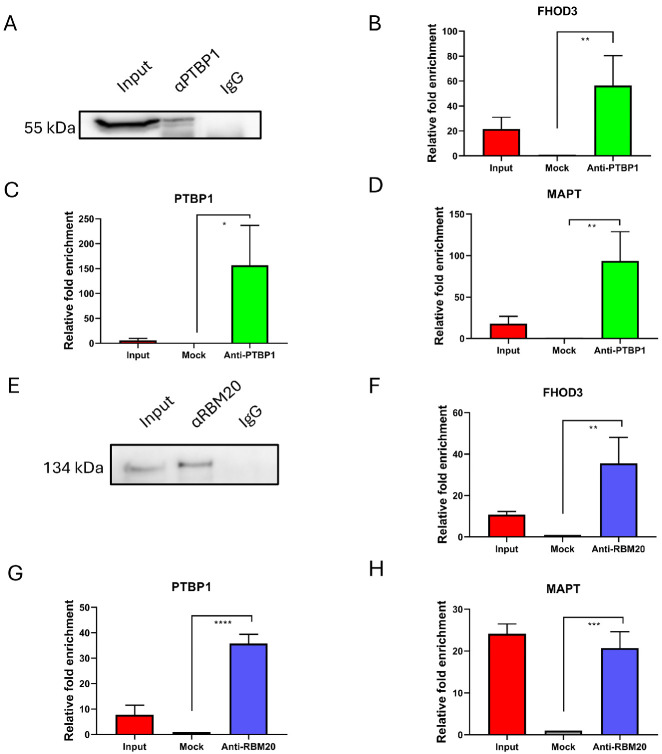
Analysis of the direct involvement of PTBP1 and RBM20 in *MAPT* mRNA regulation by RIP assay. (**A**) Western blot analysis showing the presence of PTBP1 protein in both the input and immunoprecipitated (αPTBP1) samples and its absence in the control samples precipitated using normal rabbit IgG (IgG). (**B**–**D**) qPCR analysis of FHOD3 (**B**), PTBP1 (**C**), and *MAPT* (**D**) mRNAs in the PTBP1-immunoprecipitated fraction compared to the negative controls (mock). (**E**) Western blot analysis showing the presence of RBM20 protein in both the input and the immunoprecipitated (αRBM20) samples, and its absence in the control samples precipitated using normal rabbit IgG (IgG). (**F**–**H**) qPCR analysis of FHOD3 (**F**), PTBP1 (**G**), and *MAPT* (**H**) mRNAs in the RBM20-immunoprecipitated fraction, compared to the negative controls (mock). Input = precleared cell lysate; mock = IgG-precipitated negative control; * = *p*-value ≤ 0.05; ** = *p*-value ≤ 0.01; *** = *p*-value ≤ 0.001; **** = *p*-value ≤ 0.0001.

To validate the direct involvement of PTBP1 and RBM20 in regulating Tau mRNA processing, we performed an RNA-immunoprecipitation (RIP) assay in SH-SY5Y cells (Figure 5). Western blot analysis confirmed the presence of PTBP1 in the immunoprecipitated samples using an anti-PTBP1 antibody, while no signal was detected in the negative control (normal rabbit IgG) (Figure 5A). The relative enrichment of transcripts in the PTBP1-immunoprecipitated fraction was then assessed by qPCR (Figure 5B–D). Significant enrichments of established PTBP1 targets, including FHOD3 and PTBP1 itself, were used as positive controls (Figure 5B,C) [27]. Notably, Tau mRNA was significantly enriched in PTBP1-immunoprecipitated samples compared with negative controls (Figure 5D).

RIP was subsequently performed to examine direct interactions between RBM20 and Tau mRNA. The presence of RBM20 in the immunoprecipitated complexes was confirmed by Western blot (Figure 5E). The qPCR analysis of the co-precipitated RNA demonstrated significant enrichment of FHOD3, a previously characterized RBM20 splicing target (Figure 5F) [27]. Importantly, both PTBP1 and MAPT transcripts were significantly enriched in RBM20-immunoprecipitated samples relative to negative controls (Figure 5G,H). These findings provide the first evidence of a direct interaction between RBM20 and Tau mRNA, reinforcing the role of this tissue-specific RBP in regulating Tau alternative splicing. Furthermore, we showed binding of RBM20 to PTBP1 mRNA, suggesting a role of RBM20 in regulating PTBP1 alternative splicing.

### 2.5. RBM20 Is Differentially Expressed During SH-SY5Y Differentiation into Neuronal-like Cells

**Figure 6:**
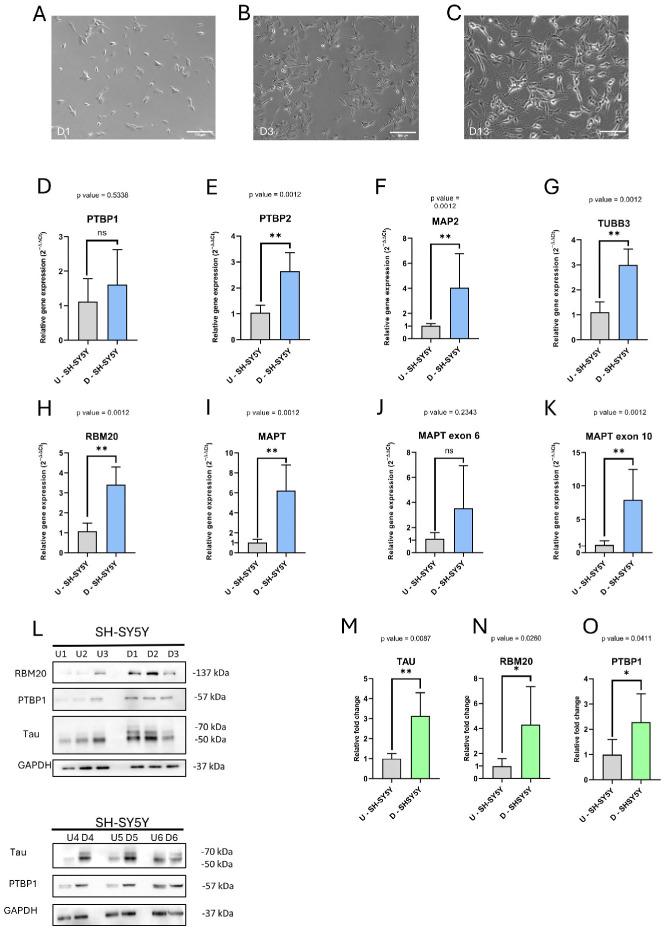
Expression analyses in SH-SY5Y after differentiation. (**A**–**C**) Morphological changes in SH-SY5Y cells during the differentiation: (**A**) Day 1, one day after seeding; (**B**) Day 3, after 2 days of ATRA treatment, short neurites can be observed; (**C**) Day 13, last day of differentiation, long neurites can be observed. (**D**–**K**) qPCR analyses to test specific gene expression after 13 days of differentiation: overexpression of *PTBP2*, *MAP2*, *TUBB3*, *RBM20* and total *MAPT* (**E**–**I**) confirms the differentiation of SH-SY5Y in neuron-like cells; (**J**) qPCR analysis of MAPT mRNA with exon 6 inclusion and (**K**) qPCR analysis of MAPT mRNA with exon 10 inclusion in differentiated cells. All the targets were normalized using GAPDH as a housekeeper. (**L**) WB analysis of PTBP1, RBM20, and Tau proteins in undifferentiated and differentiated cells. After 13 days of differentiation, bands corresponding to the higher-molecular-weight isoforms of Tau, relative to exon 10 inclusion (4R), can be visible according to the literature [31]. (**M**–**O**) Quantification of WB analyses shown in (**L**). U-SH-SY5Y = undifferentiated SH-SY5Y, and D-SH-SY5Y = differentiated SH-SY5Y. For qPCR, U-SH-SY5Y N = 7, D-SH-SY5Y N = 6; for WB, U-SH-SY5Y N = 6, D-SH-SY5Y N = 6. ns = *p*-value ≥ 0.05; * = *p*-value ≤ 0.05; ** *p*-value ≤ 0.01.

We further analyzed the expression of RBM20 during differentiation of the neuroblastoma cell line SH-SY5Y. RNA and proteins were extracted and analyzed after 13 days of differentiation via all-*trans* retinoic acid (ATRA) and brain-derived neurotrophic factor (BDNF), and compared to undifferentiated cells. Morphological changes, indicative of differentiation, were verified on the third and 13th day compared to undifferentiated cells (Figure 6A–C). The qPCR analysis showed a significant increase in total *MAPT, PTBP2, TUBB3*, *MAP2*, and *RBM20* expression (Figure 6E–I), as expected for differentiated neuronal-like cells. A significant increase in the *MAPT* exon 10 inclusion isoform and a non-significant increase in the *MAPT* exon 6 inclusion isoform were also observed in differentiated cells (Figure 6J,K). Western blot analysis was performed on a total of six replicates (undifferentiated N = 6, differentiated N = 6; Figure 6L). In the case of differentiated SH-SY5Y cells, bands corresponding to the Tau isoforms with higher molecular weights were observed, suggesting the presence of the mature Tau isoform pattern, according to the literature [31] (Figure 6M–O). The densitometric analysis showed a significant increase in RBM20, total Tau, and PTBP1 after differentiation. Unexpectedly, Tau exon 6 and 10 exclusion isoforms did not differ between undifferentiated and differentiated cells, suggesting that in neuroblastoma cells, the splicing machinery driven by deregulated PTBP1 may affect Tau isoform expression.

### 2.6. Tau and RBM20 Are Differentially Expressed During Brain Organoid Development

In order to analyze Tau and RBP expression in more sophisticated cellular models able to recapitulate in vivo neurodevelopment [32], 3D cerebral organoid models were generated from the XFiPS cell line.

**Figure 7:**
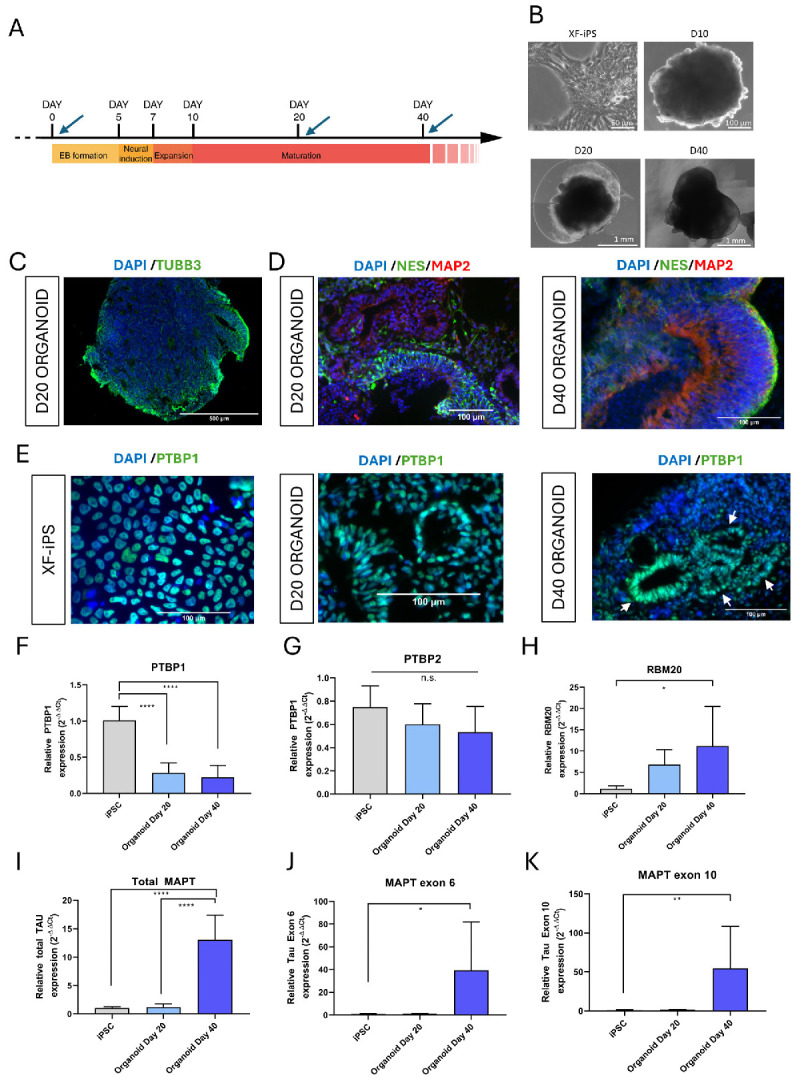
Analysis of the differential expression of PTBP1 and RBM20 during brain organoid development. (**A**) Schematic timeline representing the different stages of human brain organoid generation using the STEMdiff^™^ Cerebral Organoid Kit. Blue arrows indicate the time points of sample collection. (**B**) Representative brightfield images of XF-iPS cells and organoids after 10 (D10), 20 (D20), or 40 (D40) days of differentiation. (**C**–**E**) Immunostaining results show the immature neuron marker TUBB3 (green) at day 20 (**C**); the neural progenitor marker Nestin/NES (green), both at day 20 and day 40 and the mature neuron marker MAP2 (red) at day 40 (**D**); the neural progenitor marker PTBP1 (green) in iPS cells and organoids at day 20 and day 40 (**E**). (**F**–**K**) qPCR analysis of PTBP1 (**F**), PTBP2 (**G**), RBM20 (**H**), Tau transcripts (**I**), Tau exon 6+ (**J**), and 10+ (**K**) isoforms, using RPLP0 as housekeeping gene. * = *p*-value ≤ 0.05; ** = *p*-value ≤ 0.01; **** = *p*-value ≤ 0.0001; n.s. = not significant; EB = embryoid body; D20 = organoid after 20 days of differentiation; D40 = organoid after 40 days of differentiation.

Cerebral organoids were generated using an unguided approach, as previously described (Figure 7A) [32,33,34]. Organoids were regularly monitored throughout differentiation, displaying appropriate morphology and progressive increase in size, consistent with reported morphological phenotypes [32] (Figure 7B).

Evaluation of neural differentiation was performed by immunofluorescence staining and fluorescence microscopy using markers of neural progenitors (Nestin/NES; PTBP1), immature neurons (TUBB3), and mature neurons (MAP2) (Figure 7C–E). As shown in Figure 7C, TUBB3-positive immature neurons were detectable by D20, along with structures resembling neural rosettes. NES-positive neural progenitors were observed at both D20 and D40 (Figure 7D, in green), while MAP2-positive mature neurons became evident by D40 (Figure 7D, right panel, red). Expression of PTBP1, associated with stemness maintenance [19,35,36,37], was also examined. As shown in Figure 7E, XF-iPS cells expressed PTBP1. Upon generation of organoids, PTBP1 expression was maintained by most cells at D20. In contrast, PTBP1 expression at D40 was restricted to specific regions displaying neural rosette-like morphology (Figure 7E, white arrows), in agreement with previous reports [38,39]. Together, these data confirmed the neural phenotype of the cerebral organoids.

Having further confirmed the increased expression of the mature neuron marker MAP2 in organoids by qPCR (Figure S3), the expression levels of *PTBP1* and *RBM20* and Tau isoforms were analyzed by qPCR in samples from undifferentiated XFiPS cells, D20, and D40 organoids (Figure 7F–K). *PTBP1* expression was significantly reduced in both D20 and D40 organoids compared with undifferentiated cells (Figure 7F). *PTBP2*, a neuronal paralog of PTBP1, did not show significant changes across conditions, whereas *RBM20* expression was significantly increased in D40 organoids (Figure 7G,H). Total Tau transcript levels were significantly upregulated at D40 relative to both undifferentiated and D20 samples (Figure 7I), consistent with neuronal differentiation. Isoform-specific qPCR analysis further demonstrated the presence of transcripts containing Tau exons 6 and 10 in D40 organoids (Figure 7J,K).

Altogether, these analyses demonstrated that the brain organoids recapitulate the expression of Tau isoforms during differentiation, as well as that of the PTBP1 ribonucleoprotein, thus representing a valuable model for studying RBP expression dynamics and alternative splicing regulation during neuronal differentiation. Furthermore, this is the first report underlying an increased RBM20 expression during neuronal differentiation, suggesting a possible role for this protein in neural development.

## 3. Discussion

Tau protein is implicated in a wide range of cellular processes, including microtubule stabilization, axonal transport, DNA protection, regulation of gene expression, miRNA activity, and protein synthesis [4]. The involvement of Tau in different biological functions can be partially explained by the extensive alternative splicing of the *MAPT* gene, which generates more than 16 different isoforms [1,4]. Proper isoform balance is crucial for CNS homeostasis, since its disruption has been widely associated with Tau hyperphosphorylation and aggregation, leading to the onset of the so-called tauopathies [6,40]. In particular, a 1:1 Tau 3R/4R ratio is typical of the healthy adult brain, whereas alterations in exon 10 alternative splicing that affect this balance have been linked to neurodegeneration [5,41,42]. Increased activity of kinases, such as GSK3β and CDK5, together with reduced activity of phosphatases, especially PP2A, promotes Tau detachment from microtubules and enhances its aggregation propensity [43,44].

In this context, we have pointed out our interest in exon 6 alternative splicing, a relatively understudied event, due to evidence suggesting a neuroprotective role for the Tau 6p and 6d truncated isoforms. In the cerebellum, Tau 6d isoforms are as abundant as full-length Tau but are absent in neurofibrillary tangles, potentially explaining why the cerebellum is not affected by Tau pathology in Alzheimer’s disease [1]. Moreover, Tau 6p and 6d have been shown to inhibit Tau polymerization in vitro [9]. However, exon 6 is contained within the PRR and includes multiple putative phosphorylation sites [4].

Beyond these CNS variants, *MAPT* complexity is further exemplified by ‘Big-Tau’ (~110 kDa). This isoform, defined by the inclusion of the large exon 4a, is the predominant form in the peripheral nervous system (PNS) and is found in neurons projecting to the periphery. In rodents, Big-Tau expression follows a specific developmental trajectory, as it increases postnatally [45]. Notably, although extensively characterized in rats and mice, evidence in humans remains largely based on genomic analyses. This double-size isoform is thought to be involved in stabilizing the mature axonal cytoskeleton. The limited number of phosphorylation sites within exon 4a suggests that Big-Tau may be less prone to the pathological aggregation typically observed in CNS tauopathies [1,4].

RNA-binding proteins (RBPs) are responsible for modulating alternative splicing events and maintaining isoform equilibrium [46]. For this reason, characterizing their role in the regulation of *MAPT* splicing may contribute to developing RNA-based therapies and identifying novel targets in tauopathies [40].

In this study, we investigated the role of two splicing factors, PTBP1 and RBM20, in regulating Tau isoforms expression. Using a combination of minigene constructs and advanced cellular models, including brain organoids, we demonstrated that PTBP1 and RBM20 act similarly in regulating Tau exon 6 and 10, promoting their exclusion from the final transcript. RIP assays further provided evidence of PTBP1 and RBM20 interacting with total Tau mRNA, thereby establishing a new connection between this tissue-specific RBP and neuronal RNA splicing regulation. Finally, 2D and 3D cellular models, such as the SH-SY5Y cell line and cerebral organoids, confirmed the involvement of RBM20 in neuronal differentiation and its correlation with Tau alternative splicing regulation.

Previous studies have already identified PTBP1 as a key regulator of Tau splicing in monkey kidney cells (COS cells), showing that its overexpression promotes skipping of exons 6 and 10 using minigene constructs. The approach used in these studies relied on cloning extended intronic regions flanking exons 6 and 10 into the vectors, focusing on the importance of upstream and downstream sequences in splicing regulation [3,47,48,49]. The 5′ splice site of exon 10 was demonstrated to influence the splicing mechanism; in fact, mutations disrupting the stem-loop structure are able to increase exon 10 inclusion in patients affected by FTDP-17 [50,51]. Along the same lines, another work of significant interest utilized a minigene construct spanning from exons 9 to 11 to demonstrate that a minimal limiting length of intron 10 is required for correct splicing [52]. However, all these studies lacked prior in silico analysis, and therefore, the exact cis-regulatory elements driving exon exclusion remained undefined. In contrast, our work integrates bioinformatic analysis with experimental validation, enabling the identification of clusters of polypyrimidine-rich motifs, particularly CU-rich hotspots, within restricted flanking intronic regions (200–600 bp). By selectively incorporating these putative *cis*-acting elements into our minigene constructs, we start to identify the sequences involved in PTBP1 and RBM20-dependent regulation, providing a more detailed framework for understanding Tau splicing. Based on the obtained bioinformatic results, we propose a molecular mechanism by which these two proteins target intronic regulatory elements within introns 5/7 and 9/11 to drive the skipping of exons 6 and 10, respectively. By binding to C/U motifs within intronic sequences, PTBP1 and RBM20 are able to form a stem-loop secondary structure that leads to exon skipping.

Our results in HEK293T cells are consistent with recent literature data, showing that PTBP1 overexpression in transgenic mice, expressing the full wild-type human *MAPT* gene, increases the 3R/4R ratio, whereas in vitro silencing of the protein leads to a reduced ratio [53]. Moreover, PTBP1-mediated regulation of *MAPT* splicing has also been described in cancer, further highlighting the importance of investigating the mechanisms linking PTBP1 to Tau splicing regulation across different pathological contexts [54]. Importantly, these observations reinforce the central role of PTBP1 as a regulatory factor, emphasizing the need for further investigation across multiple disease contexts, including neurodegeneration.

In contrast, RBM20 has been characterized as a heart-specific splicing factor involved in titin (TTN) splicing regulation and cardiac development [14,22,55]. RBM20 gained clinical relevance when Brauch et al. identified a cluster of pathogenic heterozygous missense mutations within exon 9, in the serine–arginine (RS) domain responsible for nuclear localization. These variants, including the R636S and P638L substitutions, were found to co-segregate with dilated cardiomyopathy (DCM) cases, establishing RBM20 as a critical regulator of cardiac function [24]. To date, 14 RBM20 mutations in the RS domain have been associated with DCM [55]. Until now, there has been no evidence for a role of this tissue-specific protein in neurons. Recent studies showed RBM20 expression in the mouse neocortex and demonstrated its splicing regulation of long neuronal pre-mRNA [29]. This evidence strongly supports our findings about the interaction between this protein and Tau transcripts. Overexpression of RBM20 was able to induce Tau exon 6 and Tau exon 10 exclusion from the mature RNA, demonstrating a possible involvement in MAPT alternative splicing. Taken together, these results highlight the largely unexplored role of Tau exon 6, paving the way for future investigation into RBM20 involvement in the onset of tauopathies.

To validate our hypothesis, we performed an RNA-immunoprecipitation assay to isolate our proteins of interest along with their associated RNA molecules. Our results strongly confirm the direct interaction between PTBP1, RBM20, and Tau transcripts, representing the first description of a physical assembly between these two proteins and *MAPT* mRNA. Notably, these findings are consistent with recent work implicating RBM20 in neuronal splicing processes, further supporting its potential role in Tau RNA regulation [29].

The role of RNA-binding proteins has been previously described in neurodegeneration [56]. From a clinical perspective, the identification of RBM20 as a regulator of Tau alternative splicing raises the possibility that its alteration may contribute to cytoskeletal deregulation associated with tauopathies.

Future studies will be required to determine whether RBM20 is differentially expressed or functionally impaired in patient-derived samples, including brain tissues or biofluids, and whether such changes correlate with disease progression or severity. In particular, the study of mutated RBM20 activity in neuronal cells might contribute to identifying diagnostic markers for neurodegenerative diseases.

Moreover, it may be worth exploring whether modulation of RBM20 activity offers novel therapeutic opportunities. Using small molecules or antisense oligonucleotides to target alternative splicing has already shown potential for treating tauopathies [57].

Furthermore, we explored the functional role of the RBPs of interest during neuronal differentiation in SH-SY5Y neuroblastoma cells. We observed an increase in *MAPT* expression, consistent with neuronal maturation, as previously described [58]. Moreover, the results of total Tau expression in Western blot analysis showed a marked change in the isoform’s band profile, according to the mature Tau isoform pattern reported in the literature [59]. We observed a significant PTBP1 upregulation upon SH-SY5Y differentiation that may be due to the neuroblastoma origin of SH-SY5Y cells [60,61], as PTBP1 increased expression has been previously reported in *MYCN*-driven neuroblastoma (NBL) cells [62]. Moreover, altered PTBP1 functionality has been associated with aberrant mis-splicing events, which can in turn promote development and progression in several types of cancer [63], further solidifying PTBP1 involvement in the establishment of cancer-specific splicing programs.

We also found RBM20 significantly upregulated after SH-SY5Y differentiation, highlighting its potential role in regulating splicing events in neuronal cells.

To better recapitulate in vivo neurodevelopment, we generated 3D brain organoids from iPSCs, which contain both progenitors and mature neurons [32]. In this system, *PTBP1* expression decreased during differentiation, in agreement with neuronal maturation, while *RBM20* expression increased. Data from the Human Protein Atlas further support neuronal enrichment of *RBM20* transcripts, and recent findings suggest its involvement in the splicing of neuronal genes such as *SEMA6D*, *DAB1*, and *MECP2* [64]. This opens new perspectives on potential neural functions of RBM20.

Tau isoform expression also changed during organoid differentiation, with increased inclusion of exons 6 and 10, consistent with developmental regulation observed in vivo [65,66]. Exon 6 inclusion, restricted to specific adult brain regions, carries phosphorylation sites that may enhance the propensity for aggregation [4,67].

Brain organoids have recently been used to model tauopathies such as Alzheimer’s Disease [68], Frontotemporal dementia [69], and Parkinson’s Disease [70,71], as well as splicing-related disorders [72,73], especially focusing on the alternative splicing of exon 10 [74,75]. However, no prior studies have examined Tau exon 6 alternative splicing in a 3D context. Our results demonstrated the feasibility of using organoids to recapitulate MAPT splicing regulation during neuronal development. Organoid technology represents a powerful tool for personalized medicine, offering patient-specific models to study Tau splicing defects while overcoming the ethical limitations of animal use.

## 4. Materials and Methods

### 4.1. Bioinformatic Analysis for RBP Binding Site Identification in Genomic Regions

The potential PTBP1 and RBM20 binding sites in the genomic regions flanking *MAPT* exons 6 and 10 were identified using FIMO (Find Individual Motif Occurrences) v5.5.6 [76]; *p*-value threshold was set at 0.001. The experimentally validated motifs of PTBP1 were obtained from the ATtRACT database [77] with a maximum quality score (Q-score = 1), while for RBM20, the most represented RNA-binding motifs in HEK293T cells were detected using CLIP-seq [78]. The selected motifs are reported in Table 1.

### 4.2. Tau Exon 6 and 10 Minigenes Plasmid Cloning

The genomic regions identified by bioinformatic analysis (see Section 4.1) were amplified via high-fidelity PCR using the Phusion^TM^ High-Fidelity DNA Polymerase (cat F530S, Thermo Fisher Scientific, Waltham, MA, USA) using sequence-specific primers (see Section 4.14), including restriction sites for XbaI and SalI enzymes at their 5′-ends. The PCR product, extracted from the agarose gel using the QIAquick Gel Extraction Kit (cat 28704, Qiagen, Venlo, The Netherlands), and the RHCglo vector was enzymatically cut using XbaI (cat R0145S, New England Biolabs, Ipswich, MA, USA) and SalI High Fidelity^®^ (cat R3138S, New England Biolabs, Ipswich, MA, USA) enzymes, according to the manufacturer’s instructions. The digested vector and PCR products were purified using the QIAquick Gel Extraction Kit (cat 28704, Qiagen, Venlo, The Netherlands), ligated using T4 DNA ligase (cat EL0014, Thermo Fisher Scientific, Waltham, MA, USA), and then transformed into chemically competent JM109 *E. coli* (cat L2005, Promega Corporation, Madison, WI, USA). Colony PCR with primers listed in Section 4.14 was used to identify positive clones that were then confirmed via Sanger Sequencing (BMR Genomics, Padova, Italy).

### 4.3. Cell Cultures

Human embryonic kidney 293 T (HEK293T) and HeLa cell lines were cultured in Dulbecco’s Modified Eagle Medium (DMEM), including 10% of Fetal Bovine Serum (FBS), L– glutamine (20 mM), penicillin, and streptomycin. Human neuroblastoma SH-SY5Y cells were cultured in DMEM: F12 1:1, 10% of Fetal Bovine Serum (FBS), L– glutamine (2 mM), penicillin, and streptomycin. The human iPSC cell line XFiPS (CVCL_C106, Cellosaurus) was cultured in NutriStem^®^ hPSC XF medium (cat 05-100-1A, Sartorius, Göttingen, Germany) on biolaminin 521 matrix (LN521-05, BioLamina, Sundyberg, Sweden). Stemness and pluripotency of the cells were checked via immunofluorescence analyses (see Section 4.12). All cell cultures were maintained in a humidified incubator at 37 °C with 5% CO_2_ and routinely tested for mycoplasma.

### 4.4. Plasmid and Cell Transfection

Two minigene vectors were generated via molecular cloning (see Section 4.2) of a fragment including exon 6 or exon 10 into the RHCglo vector [30], purchased from Addgene (plasmid #80169, Addgene, Watertown, MA, USA). Minigene T6, named pRHCglo-MAPT-E6 (Figure S1), includes a 1035 bp genomic region corresponding to exon 6 and its flanking introns, and minigene T10, named pRHCglo-MAPT-E10 (Figure S2), includes a 1078 bp genomic region corresponding to exon 10 and its flanking introns.

Overexpression analysis of the RBPs PTBP1 and RBM20 was performed using the two previously described plasmids pcDNA6.2/N-emGFP-PTBP1 (named PTBP1-GFP) and pcDNA6.2/N-emGFP-RBM20 (named RBM20-GFP), respectively [22,27].

Briefly, HEK293T cells were seeded in a 6-well plate at a concentration of 4 × 10^5^ cells/well. Different amounts of PTBP1 (0.5 µg/well, 1 µg/well) or RBM20 (0.5 µg/well, 2 µg/well) expression vector and a constant quantity of 0.5 µg/well of the minigene vectors (pRHCglo-MAPT-E6 or pRHCglo-MAPT-E10) were transfected using the TransIT-LT1 transfection reagent (cat MIR2304, Mirus Bio, Madison, WI, USA), according to the manufacturer’s protocol. After 24 h, RNA and protein samples were extracted from the cells, as described in Section 4.5 and Section 4.8.

### 4.5. RNA Extraction and Reverse Transcription (RT)

RNA was extracted using the QIAzol Lysis Reagent (cat. 79306, Qiagen, Venlo, The Netherlands), according to the manufacturer’s protocol. RNA concentration and purity were evaluated using a NanoDrop ND 1000 Spectrophotometer (Thermo Fisher Scientific, Waltham, MA, USA). Depending on the experiment, 0.5 or 1 µg of total RNA was reverse transcribed using the QuantiTect Reverse Transcription Kit (cat 205311, Qiagen, Venlo, The Netherlands).

### 4.6. RT-PCR and Densitometric Analysis

cDNA was amplified using MyTaq™ Red Mix (cat BIO-25043, Meridian Biosciences, Cincinnati, OH, USA), according to the manufacturer’s instructions. Gene-specific primers are listed in Section 4.14. Samples were visualized by GelRed^TM^ Nucleic Acid Stain (cat 41003, Biotium, Fremont, CA, USA) using the Azure 300 imaging system (cat AZI300-01, Azure Biosystems, Dublin, CA, USA). The image quantification was performed by densitometric analysis using Image Lab software v6.1 (cat 12012931, Bio-Rad Laboratories, Hercules, CA, USA). The area of each band was expressed as a fraction of the total isoform areas present in each lane.

### 4.7. Real-Time PCR (qPCR)

Real-Time PCR analysis was performed using the SensiFAST^TM^ SYBR^®^ No-ROX kit (cat BIO-98050, Meridian biosciences, Cincinnati, OH, USA) according to the manufacturer’s instructions, and specific primer pairs (see Section 4.14). Real-Time reactions were performed in a CFX Connect Real-Time PCR Detection System (cat 1855201, Bio-Rad Laboratories, Hercules, CA, USA), and relative gene expression was analyzed using the ΔΔCt method [79], using RPLP0 or GAPDH as a housekeeping gene.

### 4.8. Protein Extraction and Western Blot Analysis

To perform the protein extraction, cells were lysed with RIPA lysis buffer or directly boiled in Laemmli sample buffer 1X. For the RIPA lysis buffer protein extraction, the lysis buffer was complemented with cOmplete^TM^ proteinase inhibitor cocktail (cat 11873580001, Roche, Basel, Switzerland) and PhosSTOP (cat 4906845001, Roche, Basel, Switzerland), then incubated for 30 min on ice. Protein concentration was determined via Bradford assay; a total of 30 µg of protein was mixed with Laemmli sample buffer 4X with 10% of 2-mercaptoethanol and loaded into SDS–polyacrylamide gels.

The proteins were separated by electrophoresis and transferred onto Polyvinylidene fluoride (PVDF) membrane (cat GE10600023, Amersham Biosciences, Chalfont St Giles, UK) by wet transfer apparatus, and then incubated with the primary and secondary antibody of interest (see Section 4.15). The signal detection was performed using the WesternBright ECL kit (cat K-12045-D20, Advansta, San Jose, CA, USA). Images were taken using the Azure 300 imaging system (cat AZI300-01, Azure Biosystems, Dublin, CA, USA) and the ChemiDoc MP Imaging System (Bio-Rad, Hercules, CA, USA).

### 4.9. RNA-Binding Protein Immunoprecipitation (RIP)

RNA-binding protein immunoprecipitation (RIP) was performed on SH-SY5Y cells using the Magna RIP^TM^ RNA-Binding Protein Immunoprecipitation Kit (cat 17-700, Sigma-Aldrich, St. Louis, MO, USA), according to the manufacturer’s instructions, with minor modifications. Immunoprecipitation was done with anti-PTBP1 (rabbit, cat RN011P, MBL Life Science, Tokyo, Japan), anti-RBM20 (rabbit, cat CAU21278, Biomatik, Kitchener, ON, Canada), or host isotype control (normal rabbit IGG, provided in the kit). After immunoprecipitation, samples were collected for Western blot analysis (see Section 4.8) and RNA analysis (see Section 4.5, Section 4.6 and Section 4.7). The relative enrichment of the mRNAs of interest, in the RIP-qPCR assay, was expressed by the equation of Wang and colleagues [80].

### 4.10. Brain Organoid Generation

Human cerebral organoids were generated using the STEMdiff^TM^ Cerebral Organoid Kit (cat 08570, StemCell Technologies, Vancouver, BC, Canada), according to the manufacturer’s instructions, based on the protocol previously described by Lancaster and colleagues [32,34]. Time points of 20 and 40 days were used for qPCR analysis (see Section 4.5, Section 4.6 and Section 4.7) or for tissue immunofluorescence analyses (see Section 4.11 and Section 4.12).

### 4.11. Cells and Brain Organoid Tissue Preparation for Immunofluorescence

Cells were grown on coated glass slides, fixed in 4% paraformaldehyde (PFA), and stored at +4 °C until use. Brain organoids were fixed for 20 min in 4% paraformaldehyde (PFA) and stored at +4 °C. Organoids were embedded in Optimal Cutting Temperature (OCT) compound and flash frozen at −80 °C for 24 h, and then cryosectioned in 20 µm-thick sections, which were conserved at −20 °C.

### 4.12. Cell and Organoid Immunofluorescence and Image Acquisition/Analysis

Organoid sections were placed in a humid chamber, washed with Triton-X-100 0.5% in PBS solution, and then incubated with a blocking solution (FBS 5% in washing solution). The samples were then incubated overnight at 4 °C with the primary antibodies in the blocking solution and then incubated with appropriate secondary antibodies. Nuclei were stained for 5 min using DAPI nucleic acid stain (4′,6-diamonio-2-phenylindole, dihydrochloride, cat D1306, Thermo Fisher Scientific, Waltham, MA, USA). Samples were mounted using FluoreGuard Mounting Medium (cat FMM060, Histo-Line laboratories, Pantigliate, Italy), and slides were dried overnight and then stored at 4 °C.

Imaging analysis was performed via fluorescence microscopy using the EVOS™ M5000 Imaging System (Invitrogen, Waltham, MA, USA), acquired thanks to the Excellence Project funding. For the antibodies, see Section 4.15.

### 4.13. SH-SY5Y Differentiation

The neuroblastoma cell line SH-SY5Y was differentiated into neuronal-like cells using an already established protocol involving sequential all-trans retinoic acid (ATRA) and brain-derived neurotrophic factor (BDNF) addition, with slight modifications [81]. On day 0, SH-SY5Y cells were plated at a cell density of 1 × 10^4^ cells/cm^2^ in 6-well plates. On day 1, the normal growth medium (see Section 4.3) was replaced with Differentiation Medium 1, consisting of DMEM, 10% FBS, 2 mM L-Glutamine, Penstrep, and 10 µM ATRA (cat 207341000, Thermo Fisher Scientific, Waltham, MA, USA). The medium was replaced with fresh Differentiation Medium 1 every other day until day 6 to ensure ATRA integrity. On day 6, the medium was changed with Differentiation Medium 2, consisting of DMEM, 2 mM L-Glutamine, Penstrep, and 50 ng/mL BDNF (cat 450-02-10UG, Preprotech, Waltham, MA, USA). The medium was replaced with fresh Differentiation Medium 2 every other day to ensure BDNF integrity. On day 13, neuronal-like cells were collected for subsequent RNA extraction (see Section 4.5) and Western blot analysis (see Section 4.8).

### 4.14. Primers

The primers used for molecular cloning, RT-PCR, and qPCR are reported in Table 2.

### 4.15. Antibodies

The antibodies used in Western blot, RIP, and immunofluorescence analyses are reported in Table 3.

### 4.16. Statistical Analysis

All data are expressed as mean ± SD, and at least three experimental replicates were performed for each experiment. Statistical differences were calculated by Student’s *t* test or one-way ANOVA using GraphPadPrism v8.0.2 (GraphPad Inc., La Jolla, CA, USA). Gaussian (normal) distribution of the samples was tested by the Shapiro–Wilk test. The Kruskal–Wallis test or the Mann–Whitney test was used for the sample distribution that did not pass the normality test (alpha = 0.05). *p* ≤ 0.05 was considered statistically significant.

## 5. Conclusions

In this work, we demonstrate that RBM20, previously known for its role in cardiac tissue, is expressed in neuronal models and may be involved in the regulation of Tau alternative splicing, highlighting its potential implication in neurodegenerative diseases. Importantly, we shed light on exon 6, a poorly characterized Tau exon, and show that it represents a novel regulatory target whose functional relevance needs further investigation. Future perspectives should aim to identify the role of RBM20 mutations and dissect the contribution of exon 6 in neuronal function and Tau phosphorylation. Additionally, applying single-cell approaches to brain organoids could provide a more detailed understanding of cell type-specific Tau splicing and RBM20 activity, offering deeper insights into cerebral heterogeneity and disease mechanisms.

## Figures and Tables

**Table 1:** RBP binding motifs for PTBP1 and RBM20.

PTBP1 Binding Motifs	RBM20 Binding Motifs
UCUU	UUCUU
UCUCU	UCUUA
UCUUC	UCUUU
CUUC	CUCUU
UUCUUC	UCUUC
UCUUU	AUCUU
CUCUC	
CUCU	
CUCUCU	

**Table 2:** List of primers used in this study.

Primer Name	Gene/Region	Sequence (5′ → 3′)
**Primers Used for Cloning of Minigenes T6 and T10**		
MAPT_I5_SALI_F	MAPT	GCTGTCGACTTCAGGGGCACAGTAACA
MAPT_I6_XBAI_R	MAPT	CGATCTAGACCAAGGTCCCACCAGGTG
MAPT_I9_SALI_F	MAPT	TATGTCGACGTGCCGGTGGGTCTAGCCA
MAPT_I10_XBAI_R	MAPT	GCATCTAGAGCCTTGAACGTGTAGGAGC
**Primers Used for RT-PCR**		
TAU_F5	MAPT	CTCGCATGGTCAGTAAAAGC
TAU_R7	MAPT	CAGAGCTGGGTGGTGTCTTTG
TAU_F9	MAPT	AGACCTGAAGAATGTCAAGTCCA
TAU_R11	MAPT	TGGTTTATGATGGATGTTGCCT
GAPDH_F	GAPDH	TGAAGGTCGGAGTCAACGGATTTGGT
GAPDH_R	GAPDH	CATGTGGGCCATGAGGTCCACCAC
RSV5U	RHCglo vector	CATTCACCACATTGGTGTGC
TNIE4	RHCglo vector	AGGTGCTGCCGCCGGGCGGTGGCTG
**Primers Used for qPCR**		
PTBP1_F	PTBP1	ACCAGGCCTTCATCGAGAT
PTBP1_R	PTBP1	GTTGGGAGAGCTGTCGGTCTT
PTBP2_F	PTBP2	TCTGAGCATGTGCAGACTGG
PTBP2_R	PTBP2	GTCCCACCTGGTTGTCAGTT
RBM20_F	RBM20	GATTGGGAGAGTGAAAGTGAGG
RBM20_R	RBM20	GGATGTCTGGTTCCACGATAAA
FHOD3_F	FHOD3	GGTCATCACCGAGTGGTCTT
FHOD3_R	FHOD3	CCGCTCTATTCTCTGCAACC
TUBB3_F	TUBB3	TCAGCGTCTACTACAACGAGGC
TUBB3_R	TUBB3	GCCTGAAGAGATGTCCAAAGGC
MAP2_F	MAP2	AGGGCTTAGCAGTCCTGAAAGG
MAP2_R	MAP2	CTTCCTCCACTGTGACAGTCTG
GAPDH_F	GAPDH	GTCTCCTCTGACTTCAACAGCG
GAPDH_R	GAPDH	ACCACCCTGTTGCTGTAGCCAA
RPLP0_F	RPLP0	ACATGTTGCTGGCCAATAAGGT
RPLP0_R	RPLP0	CCTAAAGCCTGGAAAAAGGAGG
**Primers Used for qPCR of Tau Specific Isoforms**		
TAU_E6_FW	Isoform 6+	ACATCCACACGTTCCTCTG
TAU_E6_RV	Isoform 6+	TCTGAGCTACCAGGAGTGG
TAU_E10_FW	Isoform 10+	TGCAGATATTAATAAGAAGCTGGAT
TAU_E10_RV	Isoform 10+	CCGGGACGTGTTTGATATTA
TAU_E9_FW	All isoforms	CCGTCTTCCGCCAAGAG
TAU_E9_RV	All isoforms	GGTGCTTCAGGTTCTCAGT

**Table 3:** List of antibodies used in this study.

**Primary Antibodies**					
**Target**	**Host**	**Company**	**#CAT**	**WB Dilution**	**IF Dilution**
**Β3-Tubulin**	Mouse	Sigma-Aldrich	T2200	1:1000	1:200
**GAPDH**	Rabbit	Cell Signaling	2118T	1:2000	-
**GFP**	Rabbit	Genetex	GTX113617	1:1000	-
**MAP2**	Mouse	Sigma-Aldrich	M4403	-	1:200
**NES**	Rabbit	Sigma-Aldrich	N5413	-	1:200
**OCT4**	Mouse	Invitrogen	MA1-104	-	1:200
**Phospho-Tau (Ser202, Thr205), AT8**	Mouse	Thermo Fisher	MN1020	1:1000	-
**PTBP1**	Rabbit	Thermo Fisher	PA5-81297	1:1000	1:200
**PTBP1**	Rabbit	MBL	RN011P	1:1000 for RIP	-
**PTBP2**	Rabbit	Thermo Fisher	PA5-96498	1:1000	-
**RBM20**	Rabbit	Thermo Fisher	PA5-119402	1:1000	-
**RBM20**	Rabbit	Biomatik	CAU21278	1:1000 for RIP	-
**SOX2**	Rabbit	ABclonal	A0561	-	1:200
**SSEA1**	Mouse	Invitrogen	MA1-022	-	1:50
**Tau clone 12**	Mouse	Sigma-Aldrich	MAB2241	1:1000	-
**Secondary Antibodies**					
**Anti-Rabbit-HRP**	Goat	Invitrogen	31460	1:10,000	-
**Anti-Mouse-HRP**	Goat	Invitrogen	31430	1:10,000	-
**Anti-Mouse Alexa Fluor 546**	Goat	Invitrogen	A21123	-	1:500
**Anti-Rabbit Alexa Fluor 488**	Goat	Invitrogen	A31628	-	1:500
**Anti-Mouse Alexa Fluor 647**	Donkey	Invitrogen	A31571	-	1:500

## Data Availability

The data presented in this study are openly available in Zenodo repository, at doi: 10.5281/zenodo.19301198.

## Supplementary Materials

The following supporting information can be downloaded at: https://www.mdpi.com/article/10.3390/ijms27094001/s1.

## Abbreviations

The following abbreviations are used in this manuscript:

aa	Amino acid
AD	Alzheimer’s Disease
ATRA	All-trans retinoic acid
BDNF	Brain-derived neurotrophic factor
Bp	Base pair
CACNA1C	Calcium voltage-gated channel subunit α1C
CDK5	Cyclin-Dependent Kinase 5
cDNA	Complementary DNA
CNS	Central nervous system
DCM	Dilated cardiomyopathy
DMEM	Dulbecco’s Modified Essential Medium
FHOD3	Formin Homology 2 Domain containing protein 3
FTDP-17	Frontotemporal dementia with parkinsonism-17
GAPDH	Glyceraldeide-3-phosphate dehydrogenase
GFP	Green fluorescent protein
GSK3β	Glycogen synthase kinase-3 β
IP	Immunoprecipitation
MAP2	Microtubule-associated protein 2
MAPT	Microtubule-associated protein Tau
MTBD	Microtubule-binding domain
NES	Nestin
Nt	Nucleotide
NTR	N-terminal region
PD	Parkinson’s disease
PNS	Peripheral nervous system
PP2A	Protein Phosphatase 2A
PRR	Proline-rich region
PTBP1	Polypyrimidine tract binding protein 1
PTBP2	Polypyrimidine tract binding protein 2
qPCR	Quantitative polymerase chain reaction
RBM20	RNA-binding motif protein 20
RBP	RNA-binding protein
RIP	RNA immunoprecipitation
RPLP0	Ribosomal Protein Lateral stalk subunit P0
RRM	RNA Recognition Motif
RS	Serine–arginine domain
RT-PCR	Reverse transcription polymerase chain reaction
TTN	Titin
TUBB3	β3-Tubulin
UTR	Untranslated region
WB	Western blot
