# Supplementary material for: Regulation of Tau Alternative Splicing: A Novel Role for the Ribonucleoprotein RBM20
Source: Int J Mol Sci. 2026 Apr 29;27(9):4001. doi: 10.3390/ijms27094001 (PMC13164133; doi:10.3390/ijms27094001)
Supplement: Supplementary file 1 [file ijms-27-04001-s001.zip › ijms-4259888-supplementary.pdf]

Regulation of Tau alternative splicing: a novel role for the ribonucleoprotein RBM20

Andrea Corsi [1], Angela Valentino [1], Maria Giusy Bruno [1], Giacomo Menichetti [1], Francesca Belpinati [1], Marta Pérez Pereira [2,3], Maria Teresa Valenti [1], Alessandra Ruggiero [1], Elisabetta Trabetti [1], Cristina Bombieri [1,*], and Maria Grazia Romanelli [1,*]

Supplementary Materials

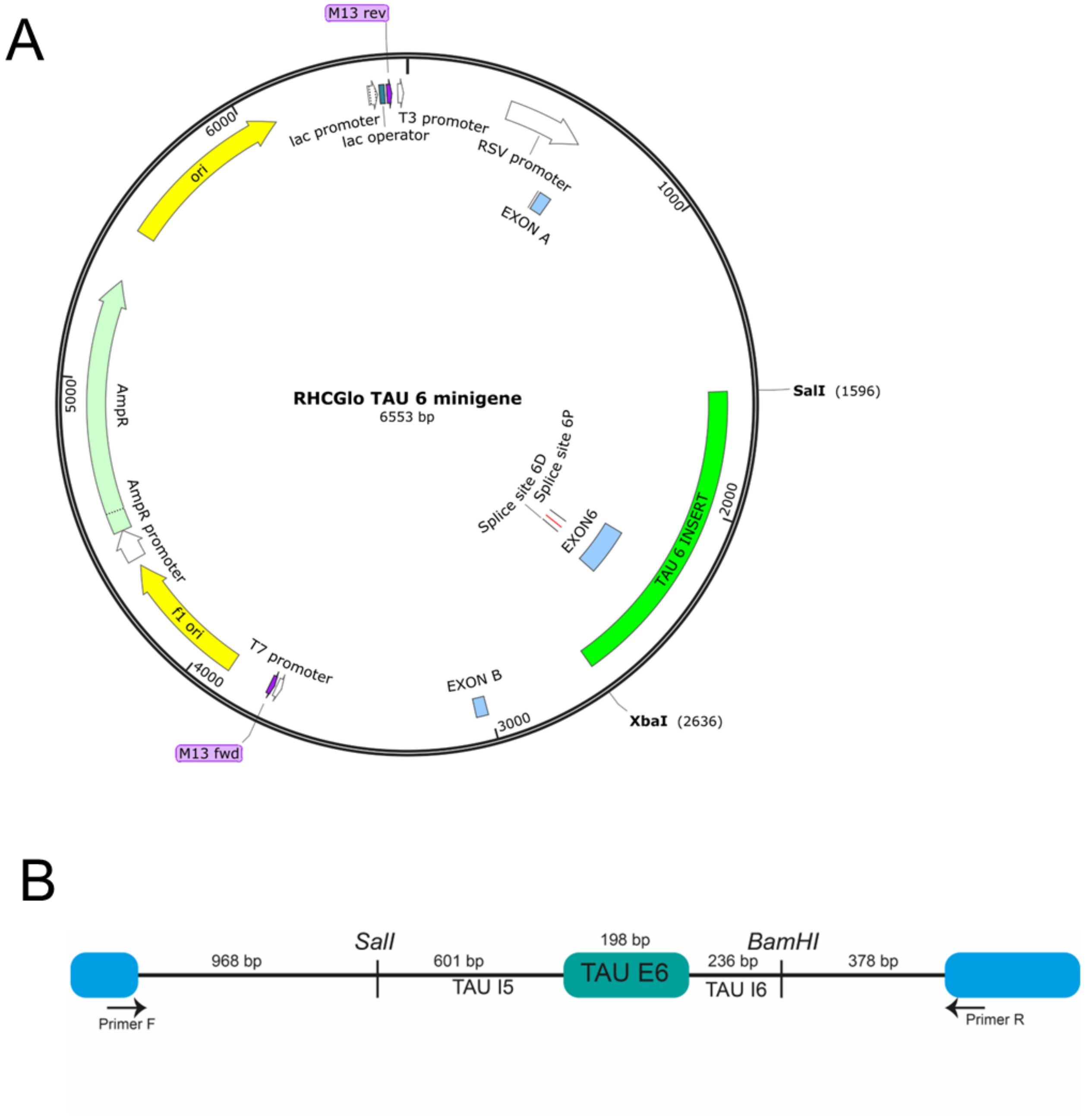

Figure S1. RHCglo Tau 6 minigene. (A) Schematic representation of pRHCglo-MAPT-E6. **(B)** Detail of the insert region. Tau exon 6 insert is flanked by 601 bp of upstream intron 5 and 236 bp of downstream intron 6 sequences, and by intronic sequences from human β-globin and chicken skeletal troponin I (sTNI). Two external artificial exons (RSV5U and TNIE4) are used for PCR primer design. The image was generated using Adobe Illustrator.

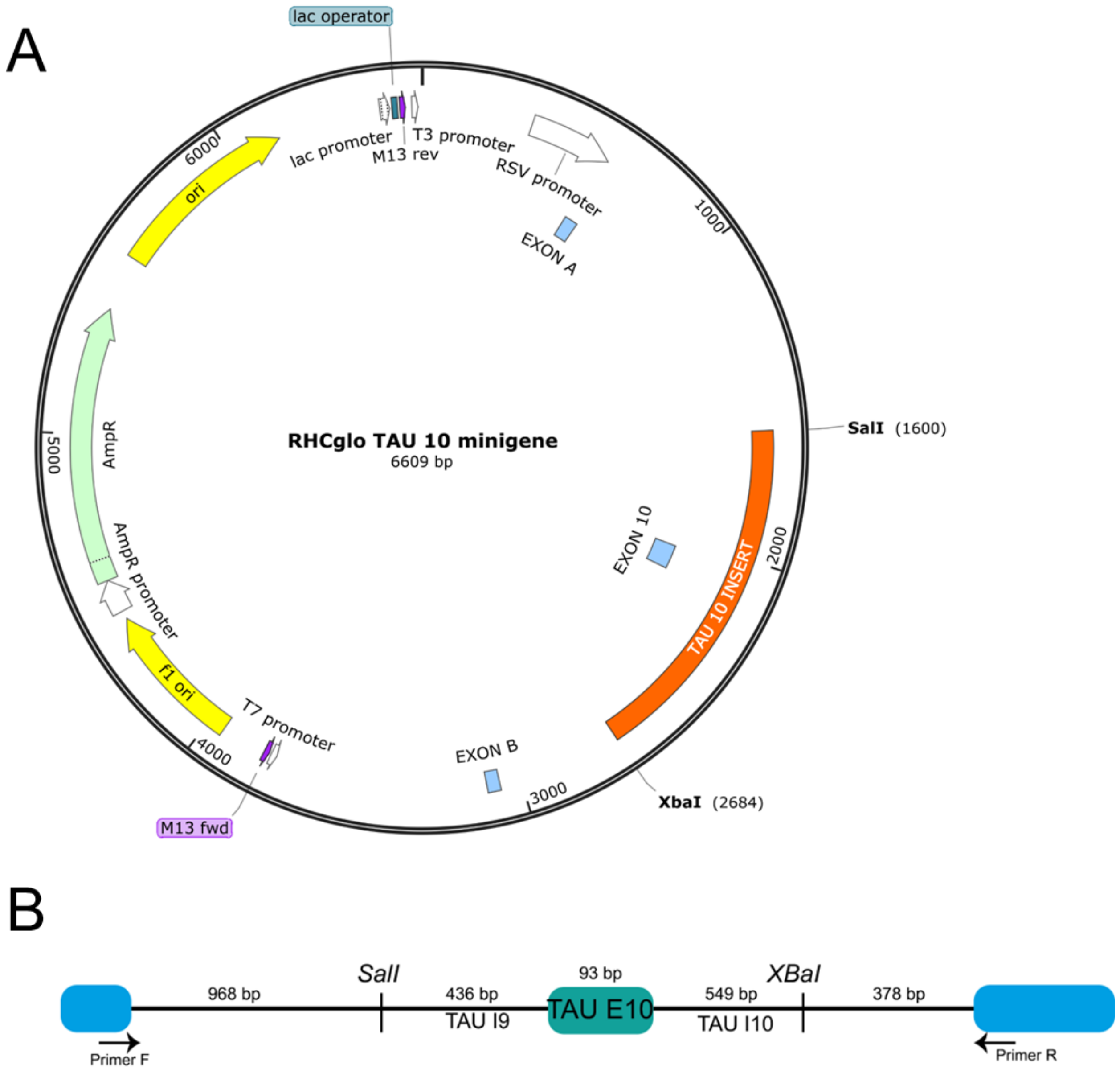

Figure S2. RHCglo Tau 10 minigene. (A) Schematic representation of pRHCglo-MAPT-E10. **(B)** Detail of the insert region. Tau exon 10 insert is flanked by 436 bp of upstream intron 9 and 549 bp of downstream intron 10 sequences, and by intronic sequences from human β-globin and chicken skeletal troponin I (sTNI). Two external artificial exons (RSV5U and TNIE4) are used for PCR primer design. The image was generated using Adobe Illustrator.

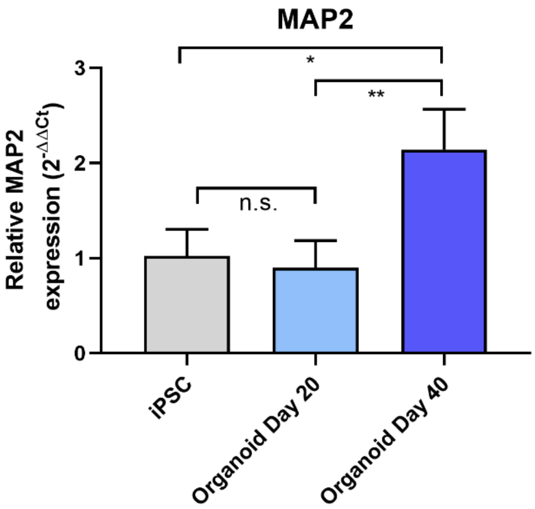

Figure S3. Analysis of MAP2 expression in HF-iPS cells and organoids at day 20 and day 40 by qPCR, using *RPLP0* as housekeeping gene. Organoids at day 40 show a higher level of mature neuron marker expression. * = p-value ≤ 0.05; ** = p-value ≤ 0.01; n.s. = not significant.
